# TANGO1 Dances to Export of Procollagen from the Endoplasmic Reticulum

**DOI:** 10.35534/fibrosis.2023.10008

**Published:** 2023-12-21

**Authors:** Carol M. Artlett, Lianne M. Connolly

**Affiliations:** Drexel University College of Medicine, Drexel University, Philadelphia, PA 19129, USA

**Keywords:** TANGO1, Fibrosis, Myofibroblast, Extracellular matrix, COPII, Systemic sclerosis

## Abstract

The endoplasmic reticulum (ER) to Golgi secretory pathway is an elegantly complex process whereby protein cargoes are manufactured, folded, and distributed from the ER to the cisternal layers of the Golgi stack before they are delivered to their final destinations. The export of large bulky cargoes such as procollagen and its trafficking to the Golgi is a sophisticated mechanism requiring TANGO1 (Transport ANd Golgi Organization protein 1. It is also called MIA3 (Melanoma Inhibitory Activity protein 3). TANGO1 has two prominent isoforms, TANGO1-Long and TANGO1-Short, and each isoform has specific functions. On the luminal side, TANGO1-Long has an HSP47 recruitment domain and uses this protein to collect collagen. It can also tether its paralog isoforms cTAGE5 and TALI and along with these proteins enlarges the vesicle to accommodate procollagen. Recent studies show that TANGO1-Long combines retrograde membrane flow with anterograde cargo transport. This complex mechanism is highly activated in fibrosis and promotes the excessive deposition of collagen in the tissues. The therapeutic targeting of TANGO1 may prove successful in the control of fibrotic disorders. This review focuses on TANGO1 and its complex interaction with other procollagen export factors that modulate increased vesicle size to accommodate the export of procollagen.

## 1. Introduction

Fibrosis, or fibrotic scarring, is a pathological process that if left unchecked can result in tissue remodeling and the formation of a permanent scar. Its mechanism is very similar to wound healing, however in contrast, wound healing is the controlled deposition of collagens. Once the wound is closed collagen is no longer secreted in abundance. Fibrosis can manifest in a single organ or can be systemic affecting many organs and tissues. Numerous different insults can contribute to the development of fibrosis. In many cases the causative agent is unknown, however, in some cases, fibrosis can be attributed to pathogens [1-3], environmental factors [4-6], genetics [7,8], autoimmunity [9-11], or cancer [12,13]. Furthermore, the risk of developing a fibrotic disorder significantly increases with increasing age [14,15]. Depending on the organ affected, there can be significant morbidity, and this can lead to death.

Much has been learned in recent years about various mechanisms that drive fibrosis, and this has largely centered on the myofibroblast. Myofibroblasts have heterogeneous origins (Reviewed in [16-18]), however, the most commonly described process is the differentiation of fibroblasts into myofibroblasts. During fibroblast to myofibroblast differentiation, there are numerous phenotypic changes to the cell. The endoplasmic reticulum (ER) becomes expanded and there is increased production of proteins, especially those belonging to the extracellular matrix family. The expanded ER is partly due to the role of ER stress in these cells [19], but it is also because there is increased folding and processing of procollagen and other large extracellular matrix proteins requiring export out of the ER. Under ER stress, the spliced form of XBP1 (X-box binding protein-1) enhances the transcription of various genes, allowing the ER to better cope with elevated levels of unfolded proteins. The spliced form of XBP1 also directly promotes ER/Golgi expansion and functions to control the secretory phenotype of the cell [20-23]. XBP1 also helps to increase the number and size of ER exit sites [24], induces a subset of proteins involved in vesicular trafficking and transport such as TANGO1 [25], and promotes Coat Protein Complex II (COPII) gene expression [26]. Overall, this helps the ER to deal with the elevated folding and processing of proteins by enhancing their export out of the ER.

## 2. Components of the COPII Vesicle

Approximately one-third of the proteins synthesized in the ER traffic to the Golgi. Many of these proteins are destined for secretion, or they reside within the various organelles in the cell. Proteins accumulate at ER exit sites for export [27]. These regions are devoid of ribosomes but are rich with COPII coat proteins and have between 2 to 6 stable COPII buds [28]. ER exit sites function as departure gates for proteins, pushing them out to other regions in the cell. Thus, the formation of the COPII vesicle for protein export is crucial to this process. The COPII vesicle includes two proteinaceous layers. These layers can deform the ER membrane to accommodate the protein cargo. The inner layer contains the Sar1-GTPase and the heterodimer Sec23/Sec24 lattice, while the outer layer comprises of a the rod-shaped heterotetramer made of Sec13/Sec31 proteins. These vesicles are assembled and disassembled as needed [29].

To start the formation of a COPII vesicle, the inner coat of the vesicle begins to assemble when Sar1-GDP is activated by the guanine nucleotide exchange factor Sec12. Sec12 loads GTP onto Sar1 causing it to bury its amphipathic helix into the outer ER membrane [30]. Sar1-GTP then engages Sec23/Sec24, which starts forming the inner coat of the vesicle. Sec23 directly interacts with Sar1. However, Sec23 also engages with Sec13/Sec31, and this causes the formation of the outer cage. In this complex, the role of Sec24 is the recruitment of various receptors for the binding of a variety of cargo. Recent studies show that the outer cage is necessary for the stabilization of the inner membrane [31]. Once the vesicle is large enough (~60–70 nm in size), it becomes unstable and the GTPase activity of Sec23 then hydrolyzes Sar1-GTP. This destabilizes the vesicle, and it buds off holding the cargo.

While overall this process is efficient for the export of smaller proteins, eukaryotic cells have evolved additional mechanisms for the export of much larger proteins such as collagens. TANGO1 (Transport ANd Golgi Organization protein 1, also called MIA3 (Melanoma Inhibitory Activity protein 3), and its paralogs in concert with other proteins have evolved to capture large proteins and expand the vesicle to hold that captured protein. The role of these proteins in the export of collagen is discussed below.

## 3. The Function of the TANGO1 Family of Proteins at ER Exit Sites

The synthesis of procollagen has been extensively studied. However, extraordinarily little is known about the export of procollagen from the ER in the context of fibrosis. The export of large proteins such as collagen is crucial under basal conditions and increased export is especially important during wound healing. Once the procollagen is folded, it must exit the ER in COPII vesicles and travel to the Golgi for further modifications. Here there is a size paradox as many of the large extracellular proteins are too big to fit into regular-sized COPII vesicles. The export of procollagen VII from the ER was first elucidated in *Drosophila* and was able to shed some light on this paradox [32]. These first fly studies found that TANGO1 and cTAGE5 (Cutaneous T-cell lymphoma-Associated antiGen 5) interacted together to create a mega-COPII transport carrier large enough to hold procollagen. More recently, other proteins have been shown to interact with TANGO1 to delay the closure of the vesicle allowing for the accommodation of procollagen.

**Figure 1.**
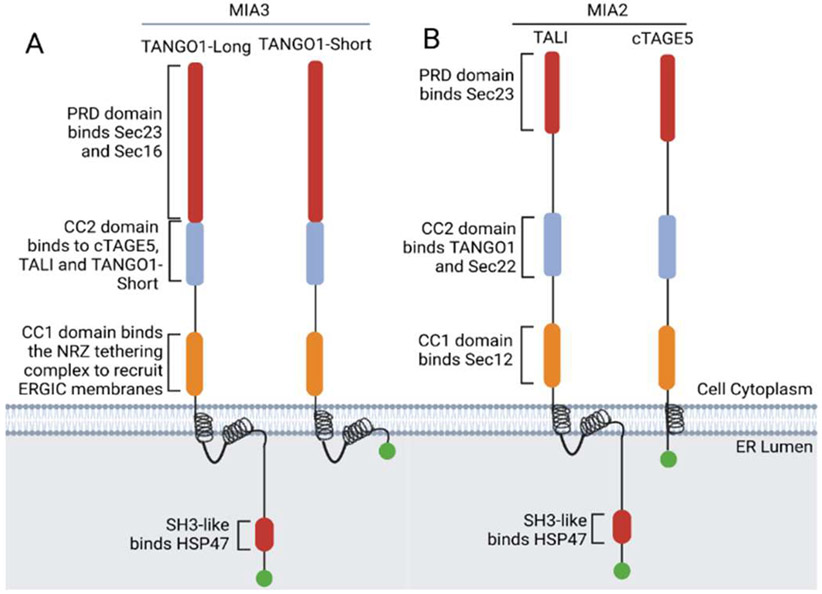
Topology of TANGO1 (Long and Short), TALI, and cTAGE5. (**A**) TANGO1 isoforms. (**B**) The TANGO1 paralog and its isoforms. The TANGO1 isoforms and paralogs embed themselves in the ER membrane using a transmembrane helix. Except for cTAGE5, there is an additional helix that partially embeds into the inner leaflet of the ER membrane to help stabilize their interaction when recruiting ERGIC membranes and procollagen. On the cytoplasmic side of the ER membrane, the proline-rich domain (PRD) binds to Sec23 and Sec16, which helps to start the formation of the COPII vesicle. The coiled-coil (CC)1 domain recruits the NBAS/RINT1/ZW10 (NRZ) tethering complex which drives the recruitment of ERGIC-53 membranes. The CC2 domain interacts with other members of the TANGO1 family; cTAGE5, TALI, and TANGO1-Short. On the lumenal side of the ER, the SH3-like domain binds HSP47 which then recruits procollagen into the complex. Figure adapted from Raote et al., 2018 [36]. Created in BioRender.com.

TANGO1 is a resident protein found in ER exit sites. It is coded on chromosome 1 and has two well-studied functional isoforms, TANGO1-Long and TANGO1-Short (Figure 1), although there are several additional splice variants with minor alterations (see Genbank). Whether these other variants have a functional role or affect protein export is currently unknown.

Saito et al. [32], showed that TANGO1 helps to orchestrate the loading of procollagen into growing vesicles. Furthermore, other studies by Saito et al. [33], demonstrate that TANGO1 also functions to organize the ER exit sites and this is a necessary step in protein export. There is controversy, however, as to how procollagen moves between the ER and Golgi. Several investigators have reported enlarged COPII vesicles are responsible while others suggest tubular-saccular structures are involved [32,34,35]. Irrespective of the mode of export out of the ER, TANGO1 plays a crucial role in this process.

### 3.1. TANGO1-Long

TANGO1-Long is a large protein consisting of 1907 amino acids. Approximately one-third of the protein is found on the cytoplasmic side of the ER. It has a transmembrane helical domain with an additional helical membrane loop that partially anchors itself into the leaflet of the ER membrane (Figure 1). On the lumenal side of the ER, TANGO1-Long has a SH3-like domain [37] that binds HSP47. It is the HSP47 that recruits procollagen into the growing vesicle. HSP47 is a critical collagen-specific ER chaperone that is upregulated in parallel with COL1A1 expression [38,39]. Studies show that the SH3 domain of TANGO1 can barely recognize and bind procollagen [40], while it is the HSP47 that strongly binds to the native type I, II, III, IV, V, VI, and X procollagens [39] and as a result recruits them into the growing vesicle.

TANGO1-Long interacts directly with cTAGE5 [34,40] and Sec12 [40]. The interaction with Sec12 helps to delay the closure of the vesicle. TANGO1-Long also has a binding site that can recruit TANGO1-S [36] and its paralog isoform TALI [41]. In keeping with the recruitment of procollagen into the growing vesicle, TANGO1-Long also recruits Sec23, allowing for the envelope to increase in size. Sec23 also co-localizes with procollagen at these sites. TANGO1-Long can also homodimerize with itself [36,42].

### 3.2. TANGO1-Short

The isoform, TANGO1-Short is 785 amino acids in length and also embeds into the ER membrane. TANGO1-Short contains the cytoplasmic domains of TANGO1-Long but lacks the HSP47 binding domain and therefore cannot bind directly to procollagen. However, TANGO1-Short is involved in procollagen export [40]. It is thought that TANGO1-Short helps to recruit the ERGIC membranes with the role of aiding in the expansion of the vesicles, allowing for procollagen to be incorporated [34,43]. TANGO1-Short competes with Sec31 for binding to Sec23. Because of this competition, there is a delay in the fission of the vesicle. TANGO1-Short has been shown to form a protein complex with cTAGE5 and Sec12 [40]. Elegant studies by Maeda et al. showed that TANGO1-Short can form homotrimers [40].

When both TANGO1-Long and TANGO1-Short are knocked down in cells, there is dissociation of Sec31 from the ER exit site [40] with subsequent destabilization of the exit site [43]. Furthermore, the depletion of both isoforms led to the decreased expression of cTAGE5 and the loss of Sec12 from the exit site [40].

### 3.3. TALI and cTAGE5

TANGO1 has a paralog located on chromosome 14. The TANGO1 duplication results in the protein TANGO1-like protein, which also has two isoforms (Figure 1). The larger isoform TALI is located in the liver and was found to be crucial in the export of apolipoprotein B [41]. Apolipoprotein B has a molecular weight of 540 kDa and is likely to be one of the largest proteins to ever be made by any cell. TALI was also found to be expressed in tissues devoid of apolipoprotein synthesis such as the lung, testes, small intestine, colon, pancreas, kidney, and prostate [41]. This suggests that TALI may be involved in the export of other large proteins. Since TALI has a HSP47 binding domain we speculate that it could also be involved in the export of collagen although to date there is no evidence for this. The short isoform of TALI, now called cTAGE5, is ubiquitously expressed and is also involved in procollagen export. The export of apolipoprotein B requires both TANGO1 and TALI, and the export of procollagen requires TANGO1 and cTAGE5.

cTAGE5 lacks a lumenal domain and therefore cannot bind directly to cargo, however, cTAGE5 interacts with TANGO1 via its CC2 domain and interacts with Sec12 via its CC1 domain. It is this binding of Sec12 and the increased recruitment of the inner COPII-coat proteins Sec23/24, concordant with the increased recruitment of outer-coat proteins Sec13/31 that helps to create larger vesicles that accommodate procollagen. cTAGE5 is the central (core) protein in cargo export. It functions as a cargo receptor via its engagement with TANGO1 (Long and Short) and it acts as a scaffolding protein for the recruitment of Sec12 [44]. The function of cTAGE5 was determined by the integration of point mutations in the protein where cTAGE5 failed to bind to Sec12, and as a result, collagen could not be exported [44].

Maeda et al. [40] reported that cTAGE5 together with Sec12 formed distinct complexes with TANGO1-Long and TANGO1-Short. Together these complexes were found to take part in the export of procollagen. The depletion of both TANGO1-Long and -Short reduced the expression of cTAGE5 without affecting the expression of Sec12. However, it was noted that Sec12 was no longer localized into the ER exit site when cTAGE5 was depleted [40]. Both TANGO1-Long/cTAGE5/Sec12 and TANGO1-Short/cTAGE5/Sec12 were found to be stable complexes within the ER exit sites. In the absence of TANGO1-Long, TANGO1-Short is sufficient to interact with cTAGE5 and Sec12 for the export of procollagen [40].

## 4. The Interaction of Other Proteins with TANGO1 for Procollagen Export

There are numerous other proteins directly or indirectly involved in the export of procollagen or other larger bulky proteins. They primarily function by delaying the closure of the vesicle so that these large proteins can be incorporated before they bud off.

### 4.1. COPII Proteins

Sec23 has two isoforms (Sec23A and Sec23B) and mutations in Sec23 have been associated with various diseases [45-47]. Mutations in Sec23A at amino acid 382 resulted in a nonsynonymous change from phenylalanine to leucine that caused craniolenticulosutural dysplasia and defects in procollagen export [46,47]. This observation was recapitulated in Sec23-KO mice, which exhibited embryonic lethality due to impaired collagen secretion [48].

Sec24 has four isoforms that can stably interact with both of the Sec23 isoforms. They have a hierarchical preference for procollagens. Lu et al. [49] showed that procollagen interacts with multiple Sec24 isoforms, but Sec24A and Sec24D played a major role in the export of procollagen. Sec24B and Sec24C can also export procollagen but to a lesser degree [49].

To date, there are no isoforms of Sec13, however, mutations within the Sec13 gene caused defects in protein secretion. Zebrafish depleted of Sec13 had developmental impairment of retinal and gut tissues associated with a defect in procollagen export [50,51]. In mammals, a Sec13-KO is embryonic lethal [52].

There are two isoforms of Sec31, Sec31A and Sec31B. Sec31A is generally expressed more than Sec31B [53], but there is differential expression of Sec31A and Sec31B in various tissues. Sec31A is highly expressed in the ovary [53] and Sec31B is highly expressed in the kidney [53]. Mutations in Sec31A result in skeletal abnormalities [54] suggesting a direct role in procollagen export.

Overall, the coupling of Sec23 to Sec24 and Sec13 to Sec31 is crucial for the export of procollagen. Sec13 stabilizes Sec31 [55]. In a study using zebrafish the depletion of Sec13/Sec31 halted the formation of the COPII vesicle causing distension of the ER [55] and retention of procollagen, while smaller cargoes were unaffected.

TANGO1 directly binds to only one of the COPII proteins. This binding would help to feed the collagen molecule directly into the growing vesicle. It directly binds to the inner layer protein Sec23, and then via this engagement Sec23 binds to Sec24. Sec13/Sec31 are recruited by Sec24. The interaction between TANGO1 and Sec23 occurs via the gelsolin-like domain of Sec23 and the repeated phosphoprotein phosphatase motifs in the TANGO1 cytosolic region [34]. Structural abnormalities and mutations in COPII proteins reported above would result in an abnormal COPII coat that cannot contain large macromolecules like collagen. This would likely explain the phenotypic abnormalities seen in mice and fish.

### 4.2. KLHL12 and CUL3

Kelch Like Family Member-12 (KLHL12), is a Bric-a-brac, Tram-track, and Broad-complex (BTB)–domain protein also involved in the export of large cargo molecules such as procollagen. Cullin-3 (CUL3)-RING ubiquitin ligase engages KLHL12 causing the monoubiquitylation of Sec31 [56]. This monoubiquination delays the closure of the vesicle, allowing for more procollagen to be incorporated. Studies have also shown the neddylation of CUL3 to be important in the process of delaying vesicle closure [57], whereas others have shown it not to be important [58]. The mild over-expression of KLHL12 leads to the formation of large COPII vesicles [56], suggesting a direct role for KLHL12/CUL3 in procollagen export. For CUL3 and KLHL12 to function properly, two calcium-binding proteins are required. These are PEF1 (Penta-EF-Hand Domain Containing 1 or peflin) and ALG2 (Alpha-1,3/1,6-mannosyltransferase) and aid in CUL3/KLHL12 recognition of Sec31. PEF1 and ALG2 dimerize to form a target-specific adaptor that causes a transient rise in cytosolic calcium levels. The increased calcium levels result in a persistence of Sec31 ubiquitylation [59,60], which in turn helps to trigger the formation of larger COPII vesicles. Calcium is a very important signaling molecule, especially in collagen synthesis [61]. Overall, this calcium-dependent regulation of CUL3/KLHL12 integrates collagen synthesis with secretion by interacting with the outer leaflet of the COPII vesicle which is composed of Sec13/Sec31 [62]. With TANGO1 anchored in the ER membrane, KLHL12/CUL3 functions to help regulate the size of the COPII transport vesicle [56].

### 4.3. Sedlin

Sedlin is a TRAPPC2 protein (TRAnsport Protein Particle C2) that aids in the targeting or fusion of ER-to-Golgi transport vesicles. Sedlin interacts directly with TANGO1 and regulates the efficient cycling of Sar1. If Sedlin is knocked down, this causes the premature closure of the vesicle leading to less incorporation of collagen and smaller vesicles [62,63].

### 4.4. Sar1

Sar1 is bound into the procollagen export machinery via its engagement with Sec12 which is directly bound by TANGO1 [64]. Sar1 has two isoforms, Sar1A and Sar1B, which share about 90% sequence identity [65]. Sar1 isoforms have a unique amphipathic amino-terminal helix but have to be activated by Sec12 to stably penetrate the bilayer to start the remodeling and deformation of the ER membrane [31]. Studies suggest that the expression of Sar1A and Sar1B are important for vesicle size and each isoform delays the closure of the vesicle at different rates. Sar1A is required for the formation of vesicles and controls membrane deformation so that vesicles can bud off when they reach the correct size. Sar1A engages with the GTPase of Sec31 more strongly than Sar1B does and therefore likely creates smaller vesicles due to the increased GTPase activity of Sec31. Therefore, for smaller cargoes, the faster kinetics mediated by Sar1A would allow for the export of these proteins. However, Sar1B binds to Sec23 more strongly than does Sar1A [66]. Sar1B is needed for the export of chylomicrons and patients with mutations in Sar1B have disorders in fat malabsorption [65,67]. In zebrafish, deficiency in Sar1B causes deficits in extracellular matrix collagen and the intracellular accumulation of procollagen [68]. Overall, these observations suggest that Sar1B is also an important protein that helps to regulate the size of the COPII vesicle for the incorporation of larger-sized proteins.

### 4.5. Sec12

TANGO1 also interacts with Sec12 in the ER exit site and this interaction also helps to delay the closure of the vesicle causing the expansion of the COPII vesicle [69]. Along with the procollagen, TANGO1 and Sec12 are exported out of the ER in the enlarged COPII vesicles and then recruited back to the ER via COPI-mediated retrograde transport [69]. HSP47 facilitates this process of retrieval of TANGO1 back to the ER. Sec12 also interacts with cTAGE5 [64] which then interacts with TANGO1 [70]. However, another study suggested that TANGO1 was retained in the ER [71].

### 4.6. Sec16

During the cell cycle, protein export from the ER is impeded [72], however, until now it was not fully understood as to how this happened. Maeda et al. recently discovered the role of TANGO1 and Sec16 in this process. During mitosis, ER exit sites are disassembled [73]. It has been shown that Sec16 is an organizer of the ER exit sites, but it is considered to be a peripheral membrane protein. Therefore, it must be recruited to the ER exit site for it to function as a scaffolding protein with other COPII components; while TANGO1-Long and TANGO1-Short span the ER membrane at ER exit sites. TANGO1-Long also interacts with Sec16 [32,70]. Maeda et al. demonstrated that TANGO1 recruits Sec16 into the ER exit site, where they interact with the organization of the site [74]. Intriguingly, during the cell cycle, TANGO1 is hyperphosphorylated and this reduces its affinity for Sec16. It is believed that this loss in Sec16/TANGO1 interaction causes the disassembly of the ER exit site [75].

## 5. TANGO1 and Fibrosis

Overall, the export of procollagen from the ER is poorly characterized in mammals and under-studied in fibrosis and other collagenopathies. The first study to report an increase and role for TANGO1 in any fibrotic disease was published by Maiers et al., [25] who elegantly showed that TANGO1 was elevated in hepatic stellate cells leading to fibrosis. They further went on to prove that the depletion of TANGO1 blocked type I collagen (COL1A1) secretion, but in their experiments the export of other matrix proteins was unaffected. Depletion of TANGO1 led to the retention of procollagen in the ER. This caused the unfolded protein response and apoptosis. They further went on to find that the unfolded protein response caused the upregulation of TANGO1 by TGFβ, and this was mediated by X-box binding protein 1 (XBP1) [25].

**Figure 2.**
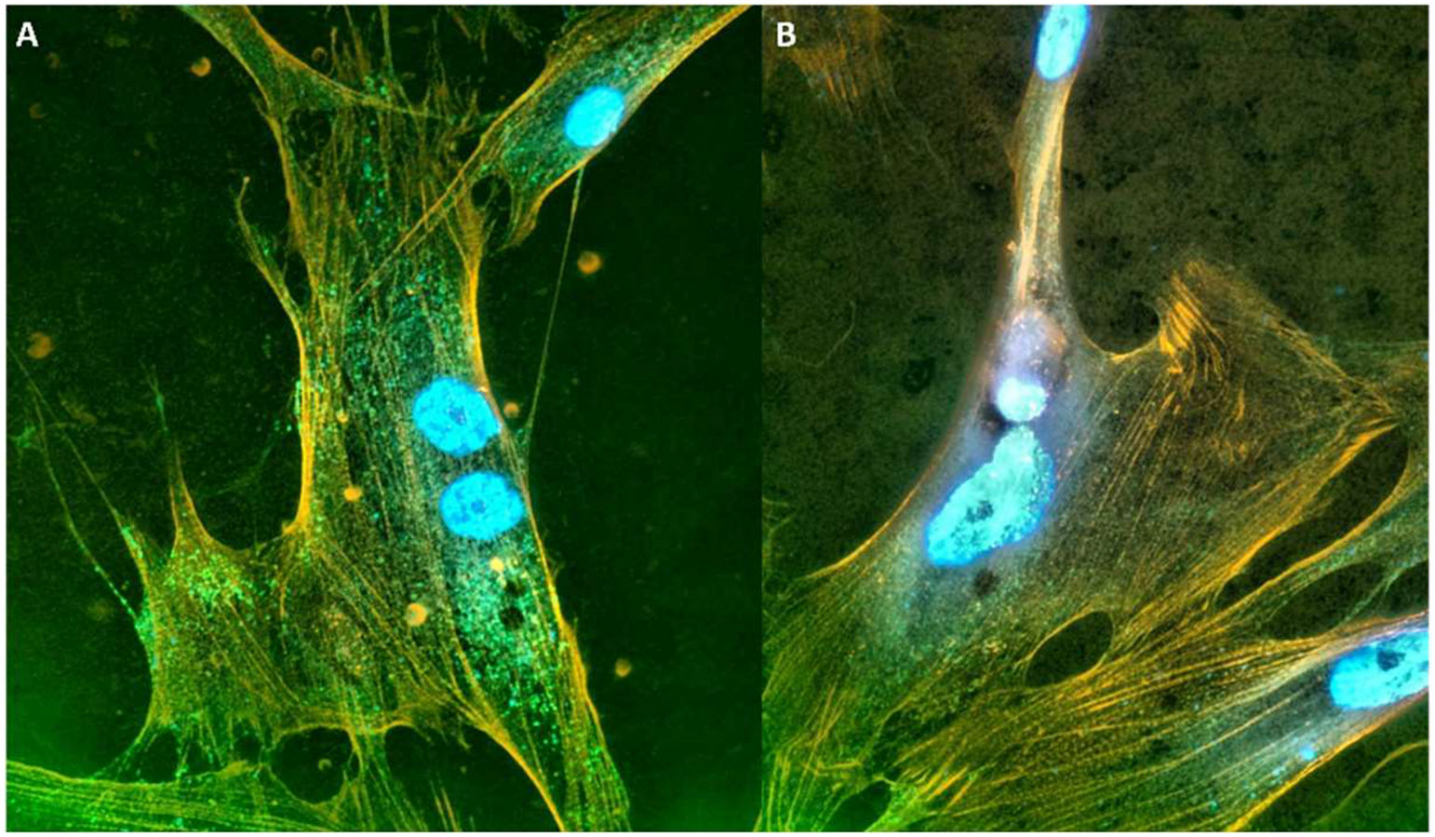
TANGO1 punctates in fibroblasts derived from SSc lesions. (**A**) Fibroblasts isolated from the lung from SSc or (**B**) normal lung tissues were stained for TANGO1 (green) and stained for f-actin (orange) using phalloidin. The nuclei were stained blue with DaPI. 1000× magnification.

Our laboratories then studied TANGO1 in systemic sclerosis [76]. Systemic sclerosis (SSc) is an autoimmune fibrotic disorder that primarily affects women. In the fibrotic lesions, there are persistently activated myofibroblasts. These activated fibroblasts drive the excessive secretion and deposition of collagen and extracellular matrix proteins in the skin, vasculature, and internal organs [77-80]. Myofibroblasts have the increased ability to secrete abundant amounts of protein and produce more collagens and other ECM proteins compared to quiescent fibroblasts. Therefore, to meet this increased secretory demand, myofibroblasts must depend on the ER to process and export the augmented protein load. We stained myofibroblasts derived from fibrotic lesions for TANGO1 and found there were increased positive punctates in fibroblasts isolated from patients with SSc (Figure 2).

**Figure 3.**
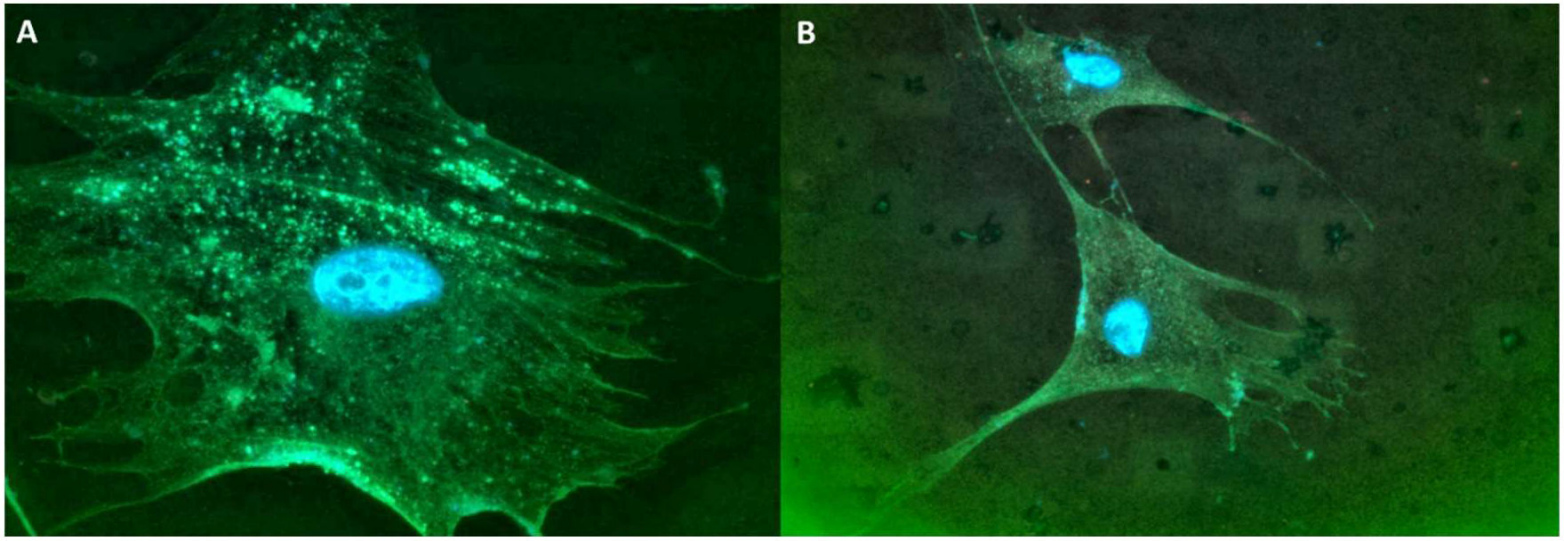
SSc fibroblasts have enlarged Sec13 punctates. SSc (**A**) and normal (**B**) fibroblasts were stained for Sec13 (green). Note the increased positive green punctates in the enlarged SSc fibroblast, compared to the normal fibroblast. 1000× magnification.

Given that there is increased procollagen export from the ER during fibrosis, we reasoned that there also had to be elevated COPII proteins. Using Sec13 as a marker for COPII proteins, we found that SSc fibroblasts also had more distinct punctates of Sec13 compared to normal fibroblasts (Figure 3).

**Figure 4.**
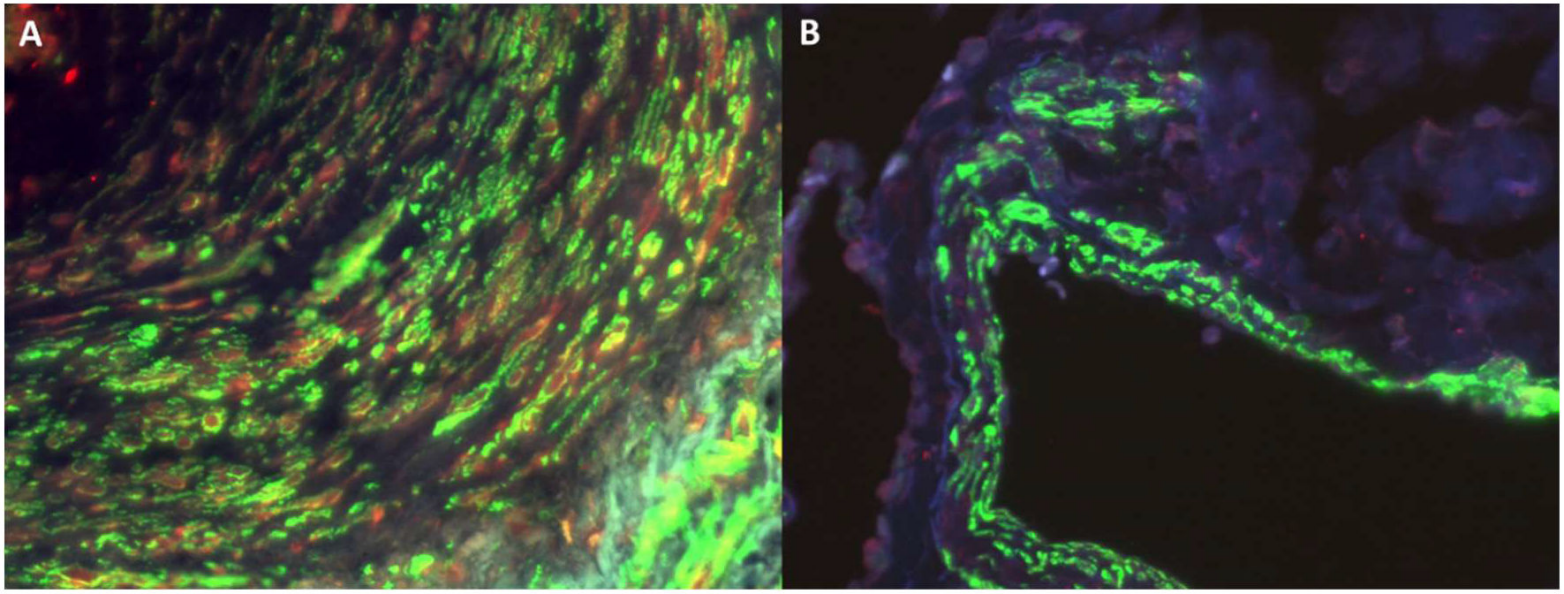
Increased expression of TANGO1 with α-smooth muscle actin in SSc vessels. (**A**) Lung tissue was isolated from a patient with SSc undergoing lung transplants or (**B**) from a normal individual who had died of unrelated pulmonary events and was dual stained for TANGO1 (red) and α-smooth muscle actin (green). Only vessels are shown. Note the increased expression of TANGO1 (red) corresponding with α-smooth muscle actin (green) in the neointima of a vessel derived from a patient with SSc (**A**), while no TANGO1 is present in the α-smooth muscle actin stained vessel derived from normal lung tissue in (**B**) [76].

We previously showed elevated TANGO1 in lung biopsies isolated from patients with SSc [76]. In the neointima of vessels from SSc patients, we saw that TANGO1 was co-localization with α-smooth muscle actin, which is suggestive of increased TANGO1 in vascular myofibroblasts (Figure 4). We especially noted this co-localization occurred in the neointima of occluded vessels (Figure 4A). In control samples, TANGO1 was not found in the vessels while α-smooth muscle actin was abundantly present (Figure 4B).

We further went on to show that TANGO1-Long and TANGO1-Short are significantly elevated in SSc fibroblasts [76] and that their expression is dependent on the activity of caspase-1. This strongly suggests the role of the inflammasome in TANGO1 expression. We further show that inhibition of the TGF-β receptor lowered both collagen and TANGO1-Long and -Short. The inhibition of the IL-1 receptor lowered TANGO1-Long and TANGO1-Short but caused the retention of collagen in the cells. In our study, we speculated that the lowered collagen expression mediated by blocking of the TGF-β receptor took pressure off the ER for the export of collagen and therefore TANGO1 expression went down. Whereas inhibition of the IL-1 receptor decreased TANGO1 expression but did not affect COL1A1 expression and therefore collagen was retained in the cell. We also found that inhibition of TANGO1 with the caspase-1 inhibitor or blockade of the TGF-β or IL-1 receptors reduced the total amounts of secreted proteins by the SSc fibroblasts suggesting that TANGO1 is also involved in the export of other proteins, not just collagen. We observed that the high molecular weight proteins were more affected than low molecular weight proteins [76]. However, we did not test to determine if the high molecular weight proteins were more likely to be retained within the cell or if these proteins also went down with the blockade of the receptors.

## 6. The Loss of Functional TANGO1 Correlates with Disease Phenotypes

Mutations in the TANGO1 gene have been associated with various diseases. In a study on clinically diagnosed Elder’s-Danlos syndrome who were negative for the classic mutations in connective tissue genes, one hundred patients were tested for mutations in TANGO1 [81]. The authors found 5 variations in the *TANGO1* gene, 4 were missense mutations and 1 was a frameshift mutation. All mutations were determined to be pathogenic. All the genetic alterations found were heterozygous and absent in the control group. The patient with the truncated form of TANGO1 had skeletal abnormalities and this was associated with scoliosis, osteopenia, brachydactyly and clinodactyly, dentinogenesis imperfecta, and mild intellectual disability. This individual also had a substantial reduction in COL1A1 secretion [81]. Another study found a homozygous synonymous substitution in the *TANGO1* gene leading to exon 8 skipping and the formation of a truncated TANGO1 protein. This truncated protein also affected COL1A1 secretion. This mutation was found in a consanguineous family [82]. Affected individuals in this family had dentinogenesis imperfecta delayed eruption of the permanent teeth, numerous skeletal abnormalities, and mild intellectual disability [82]. In another consanguineous family from India, the fetus presented with early lethality and an almost complete absence of bone formation. Whole-exome sequencing was performed and showed a novel homozygous frameshift variant of the *TANGO1* gene. This caused a premature termination codon and the complete absence of the TANGO1 protein [83].

The loss of a functional TANGO1 has been recapitulated in zebrafish and mice and confirms many of the findings seen in human diseases. When TANGO1 was depleted in zebrafish, the fish were significantly shorter in length with craniofacial defects. They did not survive to adulthood [84]. Mice lacking the *TANGO1* gene also showed chondrodysplasia and dwarfing of fetal pups. There was a shortening of the snout and limbs, compounded by tissue fragility. The complete absence of an ossified skeleton led to death [85]. Intriguingly, a new syndrome was reported in Cane Corso dogs with hereditary dental pathology that demonstrated a homozygote splice mutation that caused exon skipping in the *TANGO1* gene. The authors propose that there was some residual TANGO1 protein activity that allowed for the export of some collagen molecules and therefore the pathology exhibited in the canines was not as severe as that seen in complete knockout animals [86].

## 7. Targeting TANGO1

Targeting TANGO1 as a means to control fibrosis is an attractive option, however, there are potential caveats to this approach. We speculate that the direct targeting and blockade of TANGO1 could result in adverse events or toxicities when collagen cannot be exported, and the ER exit sites are disassembled. Although TANGO1 (Long and Short) is needed for the export of collagen it also has a functional role in the export of other proteins [76]. These will also be significantly affected. For example, TANGO1 is required for the export of apolipoprotein, and a certain amount of this protein is needed for normal mammalian health. However, more studies are warranted on the TANGO1 isoforms and paralog isoforms, certainly in the context of fibrosis, other collagenopathies, and cancer [87,88] to give a better understanding of the function of these proteins in these types of diseases. Currently, there are no TANGO1 inhibitors on the market or in clinical trials. The careful targeting of the interaction between TANGO1 and procollagen, possibly via the inhibition of the HSP47 binding domain, or with other export factors that regulate vesicle size may prove to be an effective therapeutic modality to treat a spectrum of fibrotic diseases that otherwise are difficult to control.

## 8. Conclusions

**Figure 5.**
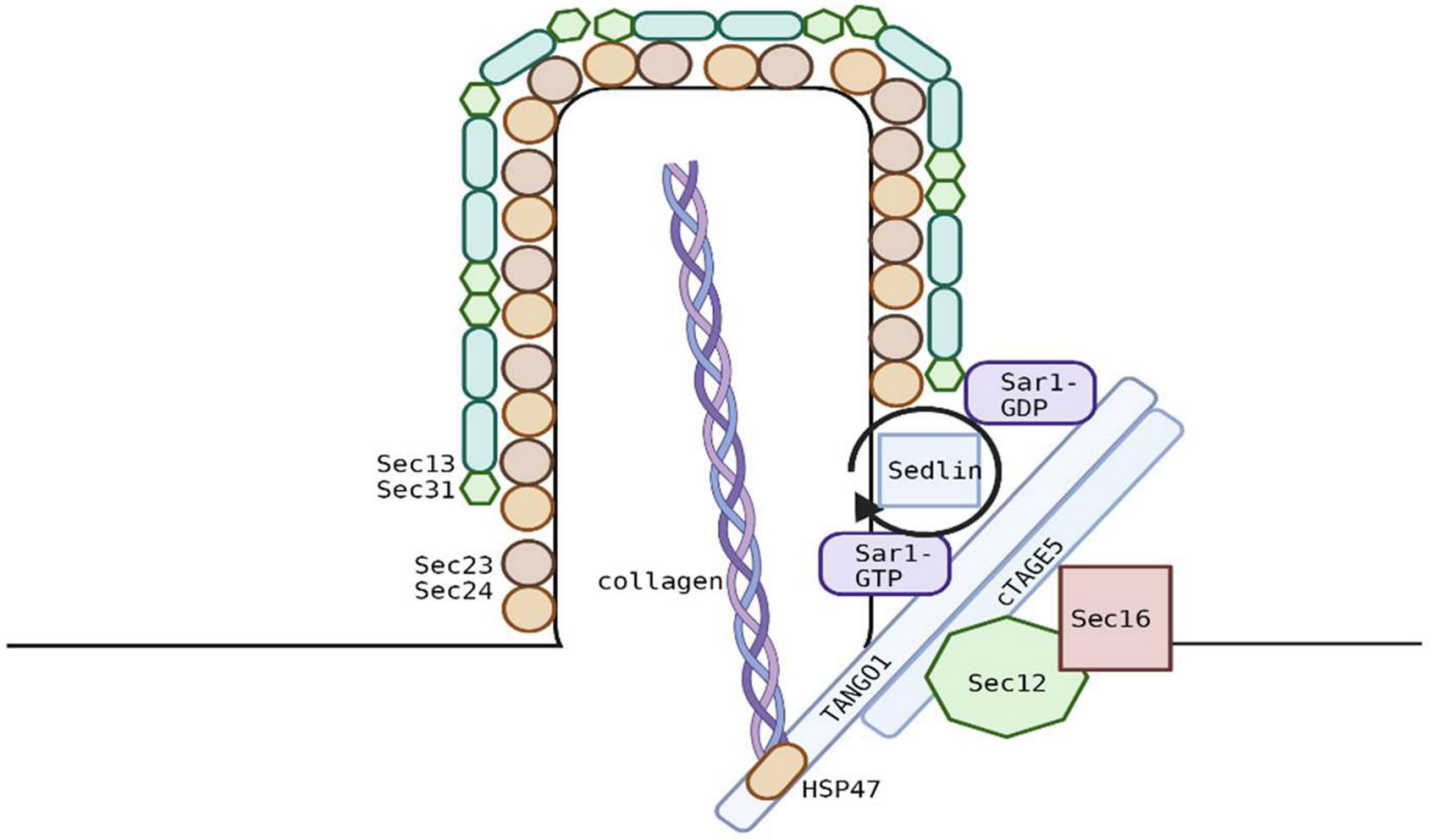
Interaction of TANGO1 with other export factors. TANGO1 binds/interacts with multiple proteins that delay the closure of the vesicle allowing for the accommodation of procollagen so that it can be exported out of the ER.

Over the last decade, studies have shown the importance of TANGO1 in regulating the export of collagen out of the ER. Overall, TANGO1 interacts with other export factors to delay the closure of the vesicle so that it can accommodate large proteins such as procollagen (Figure 5). Taken together, there is an elaborate interaction between TANGO1 and its co-factors that establish enlarged vesicles or tubules [89,90] for collagen export. Further evidence suggests that TANGO1 could be involved in the export of other cargoes as depletion or knockdown of this protein affected the secretion of other extracellular matrix proteins, while smaller cargoes were largely unaffected [76,91]. More studies on this protein are needed for us to better understand the role of TANGO1 in health and disease.
